# Toripalimab plus chemotherapy versus chemotherapy as first-line therapy for extensive-stage small cell lung cancer: a cost-effectiveness analysis

**DOI:** 10.3389/fimmu.2025.1591517

**Published:** 2025-07-01

**Authors:** Longfeng Zhang, Hongcai Chen, Huide Zhu, Zhiwei Zheng

**Affiliations:** ^1^ Department of Thoracic Oncology, Clinical Oncology School of Fujian Medical University, Fujian Cancer Hospital, Fuzhou, Fujian, China; ^2^ Department of Oncology Medicine, Cancer Hospital of Shantou University Medical College, Shantou, Guangdong, China; ^3^ Department of Pharmacy, Cancer Hospital of Shantou University Medical College, Shantou, Guangdong, China

**Keywords:** cost-effectiveness analysis, toripalimab, extensive-stage small cell lung cancer, partitioned survival model, incremental cost-effectiveness ratios

## Abstract

**Objective:**

The aim of this study was to evaluate the cost-effectiveness of toripalimab plus chemotherapy compared to chemotherapy alone as first-line therapy for extensive-stage small cell lung cancer(ES-SCLC) from the Chinese medical perspective.

**Methods:**

Our study utilized a partitioned survival model to estimate the costs and clinical outcomes for patients with ES-SCLC. The model incorporated direct healthcare costs and clinical outcomes.The primary outcome measures used in our analysis were quality adjusted life year (QALY) and incremental cost-effectiveness ratio (ICER). These measures were employed to evaluate the cost-effectiveness advantage of the treatment strategy by comparing it to the willingness-to-pay (WTP) thresholds. To account for uncertainties in the model results, One-way and probabilistic sensitivity analyses were conducted to assess the uncertainty of the model results.

**Results:**

The base-case analysis showed that the total cost for toripalimab plus chemotherapy was $50,918.81, while the cost for chemotherapy was $20,280.31.The combination toripalimab therapy led to a higher QALY value of 1.59 compared to 0.55 for chemotherapy. This translated into an ICER of $29,460.09 per QALY gained, which was below the WTP threshold of $40,343.68 per QALY. The results of the sensitivity analyses demonstrated that the findings were not significantly affected by changes in any of the input parameters.

**Conclusion:**

Our analysis suggests that toripalimab plus chemotherapy is likely to be a cost-effective first-line therapy for ES-SCLC compared to chemotherapy alone, based on the WTP threshold of $40343.68 per QALY.

## 1 Introduction

Lung cancer continues to be the leading cause of cancer-related deaths worldwide, posing a significant burden on public health systems (1). Small cell lung cancer (SCLC) is a highly malignant histological subtype of lung tumors, representing approximately 10-20% of all lung cancer cases (2). It is characterized by its aggressive nature and a propensity for diagnosis at an advanced stage (3). The majority of patients diagnosed with SCLC present with extensive-stage disease. This stage of the disease is associated with rapid progression and poor prognosis. Individuals with extensive-stage small cell lung cancer (ES-SCLC) experience a poor prognosis, with a 5-year survival rate of below 7% (4). Currently, the standard treatment approach for ES-SCLC involves platinum-based chemotherapy in combination with etoposide (5). However, even with the use of chemotherapy, the overall survival(OS) for patients with ES-SCLC remains limited. The median survival time ranges from 7 to 10 months and the 2-year survival rate is estimated to be only 10% to 20% (6). This suggests a critical need for the development of novel therapeutic approaches to improve outcomes for these patients. The emergence of immunotherapy in recent years has revolutionized the field of cancer treatment. Immune checkpoint inhibitors(ICIs), such as antibodies targeting programmed death-ligand 1 (PD-L1) or programmed death protein 1 (PD-1), have shown significant efficacy in ES-SCLC patients (7). Combination strategies, such as combining immunotherapy with chemotherapy or targeted agents, are currently being investigated to further enhance treatment response in ES-SCLC (8). For instance, the KEYNOTE-604 trial studied pembrolizumab combined with chemotherapy in patients with ES-SCLC. The trial showed that pembrolizumab combined with chemotherapy significantly improved progression-free survival (PFS), with a hazard ratio(HR) of 0.75, a 95% confidence interval of 0.61 to 0.91, and a p-value of 0.0023 (9). Another example is the IMpower133 trial, which thoroughly evaluated atezolizumab combined with chemotherapy for patients with ES-SCLC. The trial’s findings showed promising results. Patients treated with atezolizumab and chemotherapy experienced significantly better objective response rates and longer durations of response compared to those who received chemotherapy alone (10). A recent phase 3 randomized clinical trial, the EXTENTORCH study (NCT04012606), found that adding toripalimab to first-line chemotherapy enhanced PFS and overall survival for patients with ES-SCLC. In comparison to the placebo, toripalimab improved investigator-assessed PFS, yielding a HR of 0.67 (95% CI, 0.54-0.82; P <0.001) (11).

The EXTENTORCH phase 3 clinical trial has provided promising results for the treatment of ES-SCLC using toripalimab. However, it is important to evaluate the cost-effectiveness of adding toripalimab to the current standard chemotherapy regimen. Conducting a cost-effectiveness analysis can provide valuable insights for healthcare providers and policymakers when making decisions regarding treatment options. Conducting a cost-effectiveness analysis is essential, as it provides valuable information that can facilitate informed decision-making among healthcare professionals and policymakers (12). Therefore, the primary objective of this study is to assess the cost-effectiveness of including toripalimab in the standard chemotherapy regimen, in contrast to utilizing chemotherapy alone as the first-line treatment for ES-SCLC.

## 2 Method

### 2.1 The partitioned survival model evaluation model

**Figure 1:**
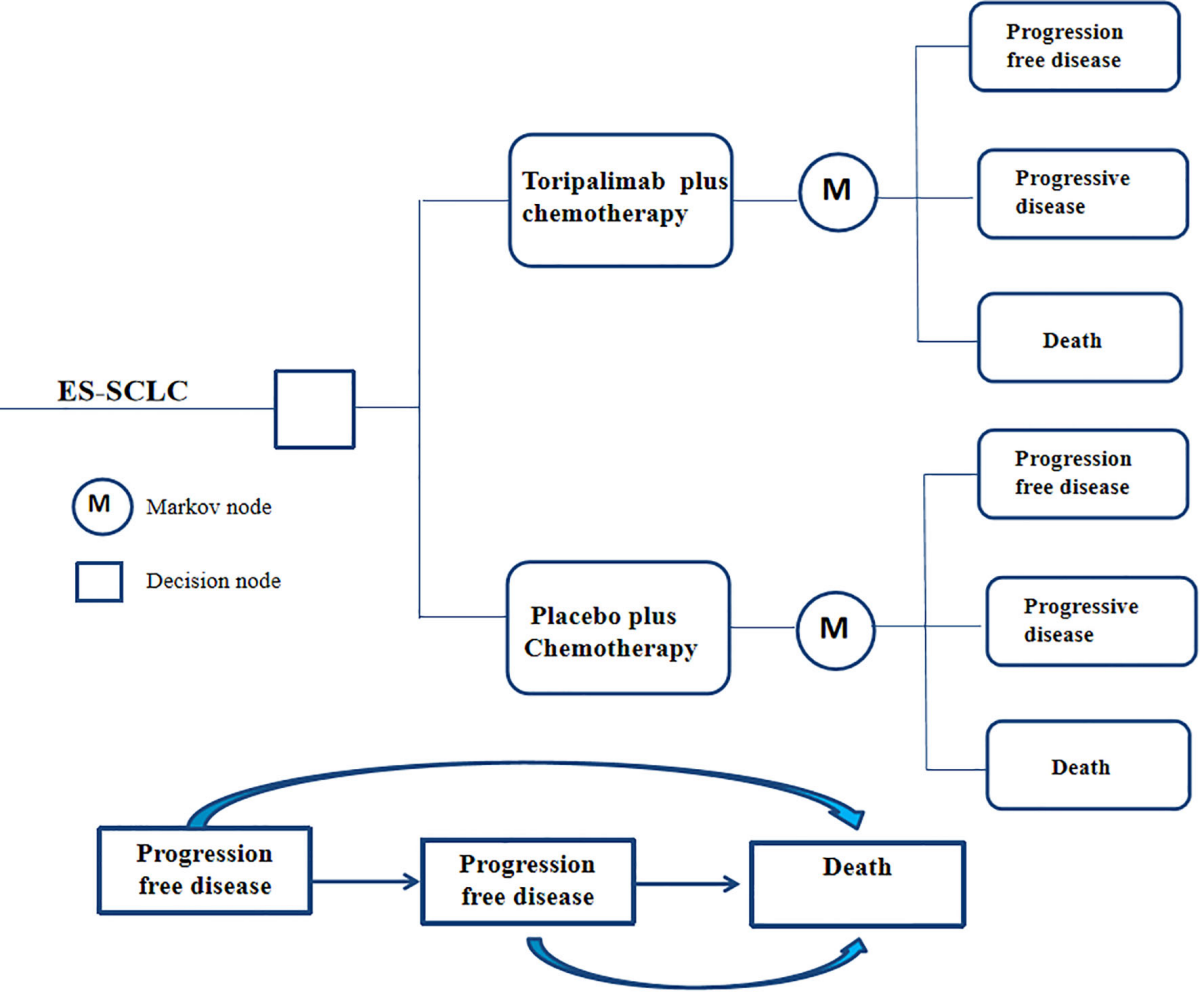
The partitioned survival model structure.

Based on the findings from the EXTENTORCH Phase 3 trial, we constructed a partitioned survival model to evaluate the cost-effectiveness of various treatment strategies for ES-SCLC. The model incorporates three distinct health states: progression-free survival, disease progression, and death, all of which are mutually exclusive. In order to account for the long-term outcomes and the relatively low 5-year survival rate associated with ES-SCLC, we have set the total time horizon of our model to 10 years. At the beginning of the simulation, patients in both treatment groups were assumed to be in a state of progression-free survival. During each treatment cycle, patients can transition to different health states based on the transition probabilities observed in the clinical trial and receive the corresponding treatment regimen assigned to them. The model structure was presented in **Figure 1**. The modeling were conducted using TreeAge Pro 2022.

### 2.2 Model transfer probability

The transition probabilities used in this model were derived from the EXTENTORCH phase 3 trial. The survival curves of the trials were then extracted using the GetData Graph Digitizer software (version2.25) and processed using R software (version 4.4.2) to reconstruct and generate simulated curves. These reconstructed curves were fitted using various parametric distributions, including weibull, log-logistic, lognormal, gompertz, exponential, and gamma distributions (13). The selection of the optimal distribution was based on two approaches. Firstly, we compared the values of the Akaike Information Criterion (AIC) and the Bayesian Information Criterion (BIC) for each distribution. Lower AIC and BIC values indicate a better fit of the distribution to the data (14). Secondly, we conducted visual examinations of the simulated survival curves to assess the suitability of the fitted distribution. The additional details regarding the data were provided in **Supplementary Table 1** and **Supplementary Figure 1**.

The analysis revealed that log-logistic distributions were the most appropriate fit for the observed survival curves in the clinical trials. In the EXTENTORCH phase 3 trial, survival and transition probabilities were directly collected from the reconstructed survival curve during the follow-up period. After the completion of the follow-up period, we used the log-logistic distribution to estimate the survival rates and transition probabilities. The calculation of the survival function was based on the time transition probability, following the equation S(t) = 1/(1 + λt^γ^) (15). The values for the parameters γ and λ were presented in **Table 1**.

**Table 1:** The reconstruct simulated parameters of the survival curve.

Parameters	OS survival curve		PFS survival curve	
	Toripalimab group	Chemotherapy group	Toripalimab group	Chemotherapy group
shape (γ)	2.26	2.44	2.27	3.26
scale (λ)	0.0020	0.0017	0.015	0.0041

### 2.3 Model clinical parameters input

Our model assumes a study population consistent with the EXTENTORCH phase 3 trial population. A total of 442 patients were deemed eligible and subsequently randomized223 patients were assigned to receive toripalimab plus EP, and 219 patients were assigned to receive placebo plus EP. The median age of the study population was 63 years, with a range of 30 to 77 years. The majority of the patients, 82.8%, were male. Inclusion criteria for the study required patients to be 18 years or older, have histologically or cytologically confirmed ES-SCLC, have an Eastern Cooperative Oncology Group performance status (ECOG PS) score of 0 or 1, have at least one measurable lesion according to the Response Evaluation Criteria in Solid Tumors (RECIST), version 1.1.

In the EXTENTORCH Phase 3 trial, patients were randomized to receive either 240 mg of toripalimab or placebo intravenously every 3 weeks. All participants also received etoposide and either cisplatin or carboplatin. The dosing regimen included intravenous administration of etoposide at 100 mg/m² on days 1, 2, and 3 of each 3-week cycle. Additionally, intravenous cisplatin (75 mg/m²) or carboplatin (area under the concentration-time curve of 5 mg/mL/min) was administered on day 1 of each cycle. After the 4–6 treatment cycles, patients continued to receive either toripalimab or placebo as maintenance therapy until disease progression or the onset of intolerable side effects. Our cost-effectiveness analysis specifically focused on the management of adverse drug reactions (ADRs), particularly those classified as grade 3–5 serious adverse events with an incidence rate exceeding 10%.

Among the patients included in the study, subsequent systemic antitumor therapy was administered to 123 patients (55.2%) in the toripalimab group and 152 patients (69.4%) in the placebo group. Based on data from clinical trials and guideline recommendations, it was assumed in our model that subsequent therapeutic interventions predominantly encompassed the administration of irinotecan in combination with cisplatin regimens.

The threshold for willingness-to-pay (WTP) in this study was set at $40343.68 per quality-adjusted life year (QALY) (16). This threshold is determined based on the criterion of three times the Gross Domestic Product (GDP) per capita in 2024 (17). All costs incurred in this study were converted from Renminbi (RMB) to United States dollars (USD) using the average exchange rate of RMB 7.12 to USD 1 in 2024 (16).

### 2.4 Model costs and utility parameters input

In this study, our main focus was on the direct medical expenses incurred during treatment, specifically those associated with the administration of toripalimab and chemotherapy agents, follow-up care, optimal supportive care, and managing adverse effects. The costs of each drug were obtained from Yaozhi.com (https://data.yaozh.com/). The median bid prices from different provinces since 2025 were collected to ensure accuracy and representativeness of the data. Additionally, we made assumptions about average patient characteristics to calculate appropriate chemotherapy dosages, including an average weight of 60 kg, a height of 160 cm and a creatinine clearance rate of 70 μmol/L.

Health-related quality of life (HRQoL) is an important measure for assessing how an individual’s health status impacts their daily activities. It is typically quantified using utility values, ranging from 0 (indicating the poorest health condition) to 1 (representing optimal health). However, no HRQoL utility values were recorded during the EXTENTORCH phase 3 trial. Therefore, we utilized utility values from the literature. The progression-free utility of 0.814 was derived from a study by Shen et al., which examined patients with advanced lung cancer. Utility values were assessed using the European Five Dimensions Health Scale (EQ-5D) and scored according to the China-specific values algorithm, resulting in the calculated utility of 0.814.Additional utility values, such as those related to disease progression and adverse effects, were obtained from a study by Nafees et al. This study involved interviews with clinical experts and patients to derive utility values and establish generalizability. The costs and utility values used in this study were presented in **Table 2**.

**Table 2:** The input parameters to the model.

Variable	Baseline value	Range		Distribution	Source
		Minimum	Maximum		
Toripalimab group ADRs (grade≥3) incidence					
Decreased neutrophil count	0.896	–	–	Beta	(11)
Decreased WBC count	0.743	–	–	Beta	(11)
Anemia	0.387	–	–	Beta	(11)
Decreased platelet count	0.306	–	–	Beta	(11)
Chemotherapy group ADRs (grade ≥3) incidence					
Decreased neutrophil count	0.894	–	–	Beta	(11)
Decreased WBC count	0.750	–	–	Beta	(11)
Anemia	0.449	–	–	Beta	(11)
Decreased platelet count	0.347	–	–	Beta	(11)
Drug cost (US dollar $)					
Toripalimab per mg	1.103	0.827	1.379	Gamma	(18)
Etoposide per mg	0.632	0.474	0.790	Gamma	(18)
Cisplatin per mg	0.213	0.160	0.266	Gamma	(18)
Carboplatin per mg	0.0853	0.064	0.107	Gamma	(18)
Irinotecan per mg	0.538	0.404	0.673	Gamma	(18)
Costs of ADRs (grade ≥3) events per cycle (US dollar $)					
Decreased neutrophil count	85.372	64.029	106.715	Gamma	(19)
Decreased WBC count	472.403	354.302	590.504	Gamma	(19)
Anemia	515.183	386.387	643.979	Gamma	(19)
Decreased platelet count	1068.487	801.365	1335.609	Gamma	(19)
Other cost					
Subsequent treatment	854.050	640.537	1067.562	Gamma	(20)
Best supportive care per cycle	359.524	269.643	449.405	Gamma	(20)
Follow-up cost per cycle	55.600	41.700	69.500	Gamma	(20)
Routine laboratory examinations per cycle	399.540	299.655	499.425	Gamma	(21)
Abdominal CT per cycle	168.070	126.053	210.088	Gamma	(21)
Utility value					
Progression-free disease	0.814	0.611	1.000	Beta	(22)
Progressive disease	0.473	0.355	0.591	Beta	(22)
Decreased neutrophil count	0.0910	0.068	0.114	Beta	(23)
Decreased WBC count	0.0910	0.068	0.114	Beta	(23)
Anemia	0.0730	0.055	0.091	Beta	(23)
Decreased platelet count	0.190	0.143	0.238	Beta	(23)
Body surface area(m^2^)	1.72	1.290	2.150	Beta	(20)
Discount rate	0.05	0.038	0.063	Beta	(17)

### 2.5 Sensitivity analysis

This study conducted one-way sensitivity analysis and probabilistic sensitivity analysis. In the one-way sensitivity analysis, we examined a range of input parameters relevant to the model and assessed their impact on the ICER. Parameters were varied up and down by 25% to capture both positive and negative effects. The resulting changes in ICER were then visually presented using tornado plots, which provide a concise overview of the parameters with the largest impact.

For conducting probabilistic sensitivity analysis (PSA), we employed Monte Carlo simulation techniques, encompassing 10,00 iterations. PSA entails incorporating the stochastic nature of uncertain parameters by utilizing probability distributions. We systematically defined each input parameter and executed the model repeatedly, such as costs were assumed to follow a gamma distribution, while utilities parameters were modeled using a beta distribution. The findings of the probabilistic sensitivity analysis were visually displayed through a scatter plot.

## 3 Result

### 3.1 The base case results

In this investigation, our findings indicate that the total costs for the toripalimab cohort were $50,918.81, while the chemotherapy cohort incurred costs of $20,280.31.Furthermore,we found that the toripalimab cohort demonstrated an incremental gain of 1.04 QALYs compared to the chemotherapy cohort. This indicates that toripalimab treatment resulted in improved patient outcomes in terms of quality of life and survival. To determine the cost-effectiveness, we calculated the ICER, which compares the additional cost of toripalimab treatment to the additional benefits gained. Our analysis revealed that the ICER for the toripalimab group was $29,460.09 per QALY. Importantly, we compared this ICER to the WTP threshold of $40,343.68.As the ICER falls below this threshold, it suggests that the toripalimab treatment protocol may be considered cost-effective in the Chinese healthcare system. The data presented in **Table 3**.

**Table 3:** The base case results.

Group	Total cost($)	QALY	Incremental cost ($)	Incremental QALY	ICER ($/QALY)
Toripalimab cohort	50,918.81	1.59	30,638.50	1.04	29,460.09
Chemotherapy cohort	20,280.31	0.55	–	–	–

### 3.2 The result of sensitivity analyses

**Figure 2:**
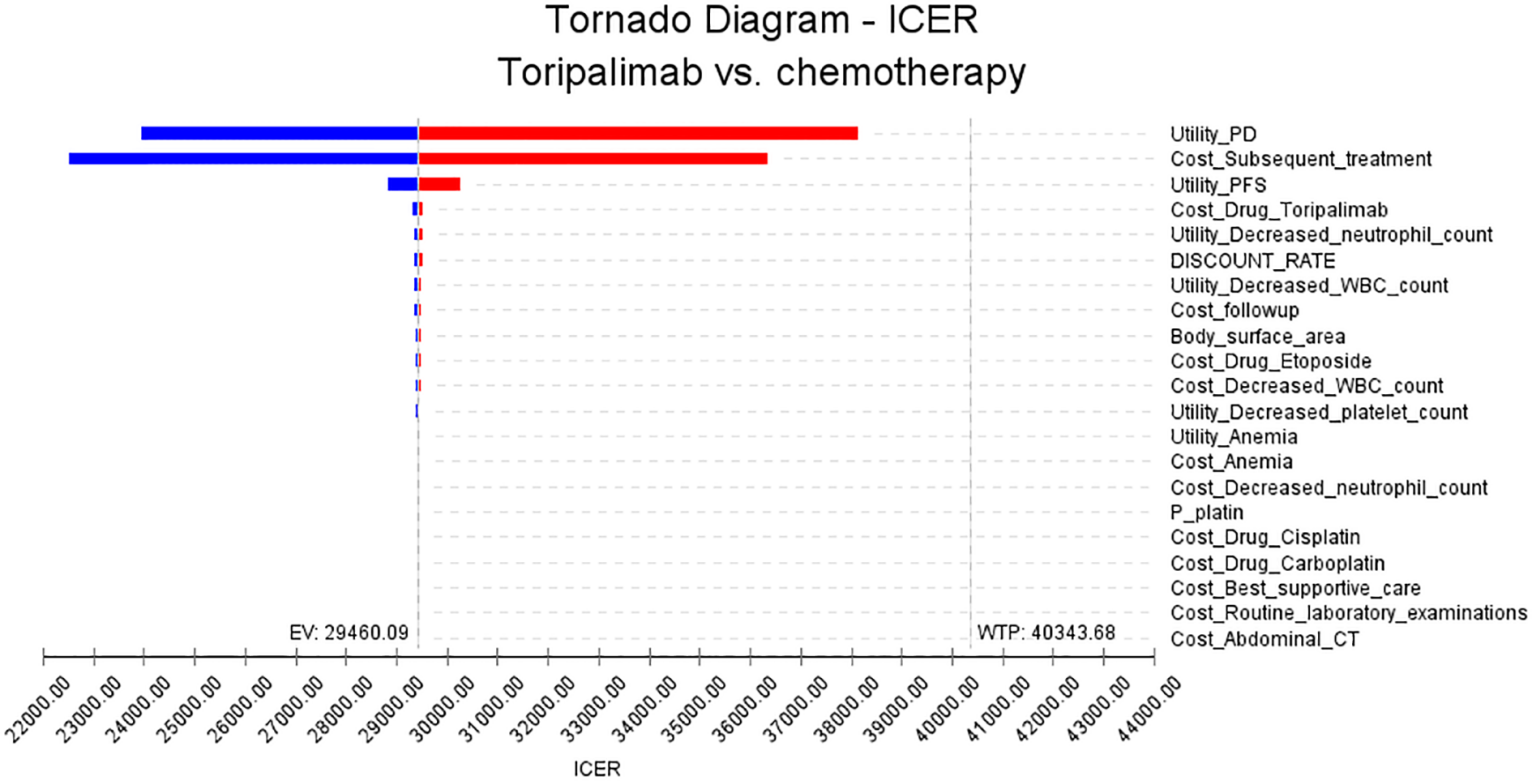
The tornado plots of the one-way sensitivity analysis.

The results of the one-way sensitivity analysis are presented in **Figure 2**. We identified several key factors that have a significant impact on the ICER in this study. These factors include the utility value assigned to progressive disease, the cost of subsequent treatments, the utility value assigned to progression-free disease, and the cost of the toripalimab drug. Our findings indicate that even when these parameters and other input variables were modified within a range of ±25%, the resulting ICER values remained consistently below the WTP threshold of $40343.68. This suggests that manipulating these parameters did not lead to substantial changes in the ICER outcomes. These results further support the conclusions derived from the base case analysis.

**Figure 3:**
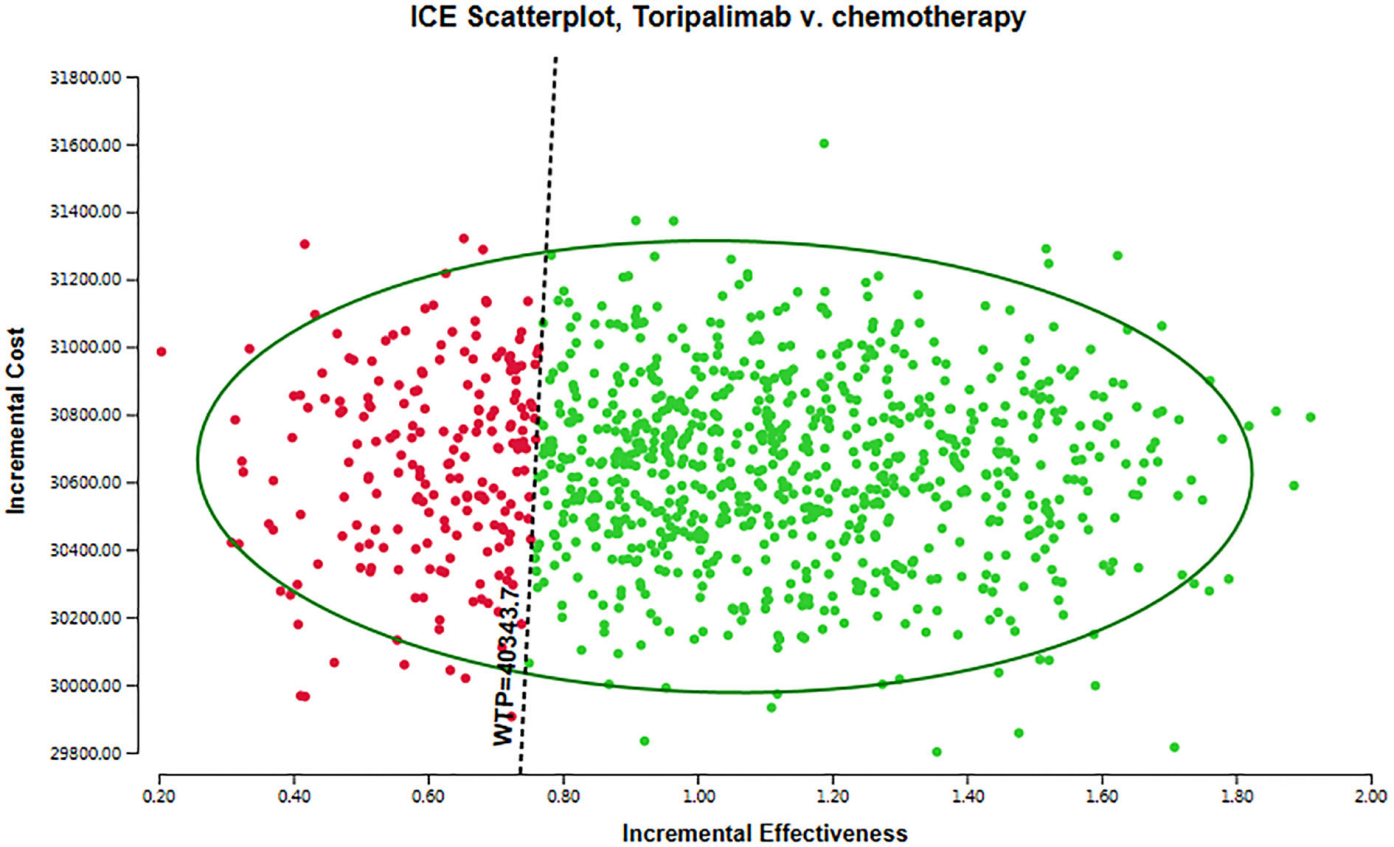
The scatter plot of probabilistic sensitivity analysis.

The results of the probabilistic sensitivity analysis are displayed in **Figure 3** as a scatter plot. The arrangement of points in different areas of the plot provides valuable insights into the likelihood of achieving various cost-effectiveness outcomes. Interventions that appear below the linear WTP threshold are categorized as cost-effective. This means that these interventions demonstrate more favorable cost-effectiveness ratios when compared to interventions located in other quadrants of the plot. The positioning of interventions below the threshold may be attributed to lower costs or improved health outcomes. It is crucial to emphasize that a WTP threshold of $40,343.68 per QALY was utilized in this analysis. Based on these results, the toripalimab group exhibited a 77.60% probability of being considered a cost-effective option in comparison to the chemotherapy group. This suggests that, according to the cost-effectiveness analysis, the toripalimab group may possess a sufficiently cost-effective advantage that could potentially enhance health outcomes.

## 4 Discussion

The EXTENTORCH trial results demonstrated a significant improvement in both PFS and OS in patients who received the combination therapy compared to those who received placebo in addition to chemotherapy. This finding highlights the potential of toripalimab as a novel treatment option for patients with ES-SCLC. Furthermore, the trial results indicate that the toripalimab combination therapy has been well-tolerated by patients, further supporting its viability as a treatment option for ES-SCLC. In addition to evaluating the efficacy and safety of a treatment regimen, it is also important to consider the economic implications of introducing a new therapy such as toripalimab. The cost-effectiveness and affordability of a treatment option are crucial factors in decision-making processes and healthcare resource allocation (24). Therefore, further research and analyses are needed to assess the economic implications of incorporating toripalimab into the treatment landscape of ES-SCLC.

In this study, the group treated with toripalimab exhibited improvement in QALYs by 1.04 compared to the chemotherapy group. However, this intervention was associated with an additional cost of $30,638.50. The ICER was $29,460.09 per QALY gained, which falls below the WTP threshold of $40,343.68.The toripalimab may be considered a cost-effective option within the Chinese healthcare system. The ICER represents the additional cost required to obtain one additional QALY. The ICER being below the WTP threshold suggests that the incremental benefits in terms of QALYs gained justify the additional costs incurred when compared to chemotherapy alone.

The sensitivity analyses were conducted to assess the robustness of the results. While no parameters led to substantial changes in the ICER outcomes, our analysis revealed several critical factors that impact the ICER in this study. One important factor is the utility value of progressive disease and progression-free disease. These values reflect the preference or quality of life associated with different health states. Assigning lower values to progressive disease or lower values to progression-free disease may result in higher ICERs, as a lower quality of life associated with these health states could decrease the cost-effectiveness of the intervention. Additionally, the cost of subsequent treatment also influences the ICER. This highlights the importance of the price of the drug in the overall cost-effectiveness analysis. A higher drug cost directly affects the ICER, making the treatment less economically viable. However, our findings indicate that even when these parameters and other input variables were varied within a range of ±25%, the resulting ICER values remained consistently below the WTP threshold of $40,343.68. This suggests that manipulating these parameters did not lead to substantial changes in the ICER outcomes and supports the conclusions drawn from the base case analysis.

The World Health Organization (WHO) recommends a cost-effectiveness threshold of one to three times the GDP per capita for the investment in one QALY (25). By taking into account the cost per QALY and its correlation with GDP per capita, the assessment of the value and feasibility of medical interventions can be enhanced (26). The connection between the cost per QALY and the GDP per capita in each country plays a crucial role in determining the cost-effectiveness WTP threshold. At this pivotal point, the cost per QALY obtained becomes the decisive factor in evaluating the worthiness and viability of interventions or policy decisions within the healthcare system (27). The notion of cost per QALY encapsulates the notion that individuals are willing to assign a specific monetary value to obtain an additional year of perfect health or to experience a significant enhancement in quality of life. WTP thresholds play a crucial role in informing decisions regarding the allocation of healthcare resources, particularly in the context of limited resources (28). These thresholds are typically used by policymakers and health technology assessments(HTA) organizations to determine whether a new healthcare intervention or technology should be funded and incorporated into the healthcare system (29). The healthcare system structure of a country impacts WTP thresholds. Countries with universal healthcare systems, where the government bears a significant portion of healthcare costs, may have lower WTP thresholds as they strive to maximize the benefits of limited resources (30). On the other hand, countries with more private or market-based healthcare systems may have higher WTP thresholds, as the decision-making may be guided more by individual preferences and WTP. Moreover, another important factor influencing WTP thresholds is the economic conditions of a country (31). In our study, according to the recommendations of China Guidelines for Pharmacoeconomic Evaluations, the WTP threshold has been set at three times the GDP (17). The WTP threshold was set at three times China’s GDP of 2024, which equates to US$40,343.68 per QALY. The ICER for toripalimab was $29,460.09 per QALY, which is below the WTP threshold of $40,343.68 per QALY. The ICER below the threshold suggests that the additional cost associated with toripalimab is justified by the improvement in health outcomes. This result indicates that the use of toripalimab as a first-line treatment for ES-SCLC may be considered cost-effective according to the established WTP threshold. However, In some high-income countries such as the United Kingdom(UK) and Australia, the use of fixed monetary thresholds, typically ranging from £20,000 to £30,000 per QALY, is a common approach in health technology assessment for determining cost-effectiveness. While a direct comparison cannot be made, it is noteworthy that the ICER falls below the £20,000 to £30,000 per QALY threshold, indicating good value for the intervention. Unlike the UK, there is no standard or official WTP threshold in the United States(US) for cost-effectiveness analysis in healthcare. Instead, researchers and institutions tend to rely on academic literature and expert opinions to inform their decision-making. In practice, many studies in the US have used a range of $50,000 to $100,000 per QALY as a reference point for cost-effectiveness analysis.

Cost-effectiveness analysis is a widely used method for evaluating the economic efficiency of healthcare interventions. It considers both the costs and benefits of different treatment options, providing important evidence for healthcare providers and policymakers when making decisions about resource allocation (32). In the field of cancer treatment, cost-effectiveness analysis is particularly relevant for comparing the cost-effectiveness of various therapies (33). There is growing interest in toripalimab due to its promising results in multiple cancer types. However, the high cost of toripalimab compared to traditional chemotherapy raises concerns about its affordability and cost-effectiveness. Based on the results from our one-way sensitivity analysis, it is evident that the ICER is impacted by changes in the price of toripalimab. Specifically, when conducting our analysis, we observed that altering the price by ± 25% resulted in different ICER. Nevertheless, it is important to note that the ICER remained consistently below the determined WTP threshold of $40,343.68.

Currently, there is limited published evidence on the cost-effectiveness of toripalimab specifically for the treatment of ES-SCLC. However, some studies have explored the cost-effectiveness analyses conducted on other cancer treatments. For instance, a recent study by Zhang et al. aimed to evaluate the cost-effectiveness of adding toripalimab to chemotherapy compared to chemotherapy alone as a first-line treatment for advanced non-small cell lung cancer patients. The findings revealed that the ICER for toripalimab was $32,237 per QALY. This suggests that the addition of toripalimab to chemotherapy has a high probability (90%) of being cost-effective in China (34). Another study assessed the cost-effectiveness of toripalimab in combination with chemotherapy for metastatic triple-negative breast cancer in China. The ICER was determined to be $16,133.18 per QALY, which suggests that toripalimab combined with chemotherapy presents a cost-effective approach for managing metastatic triple-negative breast cancer (35).

It is important to note that our analysis has some limitations. First, our study relied on certain assumptions and input parameters, which may introduce uncertainty into our findings. Sensitivity analyses were conducted to explore the robustness of our results under different scenarios. Secondly, it is important to note that the data utilized in this study are sourced from the EXTENTORCH clinical trial, which had a relatively short follow-up period. It should be acknowledged that the reconstruction of data from clinical trials can introduce certain uncertainties into our findings. Thirdly, it is important to note that our study focused solely on treatment-related serious adverse reactions of grade 3 and above, disregarding other adverse events. This could potentially introduce bias into our results when compared to real-world clinical practice, as the exclusion of certain adverse events may impact the overall cost-effectiveness assessment. However, it is worth mentioning that our sensitivity analysis demonstrated that fluctuations in costs associated with grade 3 or higher adverse events did not significantly impact the incremental cost-effectiveness ratios. Furthermore, assumptions underlying the reconstruction model are essential when reconstructing survival curves, typically involving the utilization of parametric models such as weibull, log-logistic, or other distributions. However, it is important to acknowledge that deviations from these assumptions can occur in real-world scenarios. For example, the EXTENTORCH trial may exhibit a survival pattern that deviates from the predefined parametric assumptions due to factors such as time-varying hazard rates or non-proportional hazards. These deviations have the potential to introduce systematic bias in the reconstructed survival curves. This bias can manifest as either overestimation or underestimation of survival probabilities at specific time points, impacting the accuracy and reliability of the reconstructed curves. Nonetheless, it is crucial for future studies to consider a broader range of adverse events in order to provide a more comprehensive evaluation of the cost-effectiveness of the treatment.

## 5 Conclusion

The combination of toripalimab with platinum and etoposide presents a highly promising and potentially cost-effective strategy for the initial treatment of ES-SCLC. This innovative approach not only demonstrates significant clinical efficacy but also offers a cost-effective solution for the management of ES-SCLC.

## Data Availability

The original contributions presented in the study are included in the article/**Supplementary Material**. Further inquiries can be directed to the corresponding author.
