# Supplementary material for: Toripalimab plus chemotherapy versus chemotherapy as first-line therapy for extensive-stage small cell lung cancer: a cost-effectiveness analysis
Source: Front Immunol. 2025 Jul 1;16:1591517. doi: 10.3389/fimmu.2025.1591517 (PMC12259673; doi:10.3389/fimmu.2025.1591517)
Supplement: Supplementary file 1 [file Table1.docx]

**Supplementary Table 1. Comparison of survival models distribution**

|  | AIC | | BIC | |
| --- | --- | --- | --- | --- |
|  | Toripalimab  group | Chemotherap**y** group | Toripalimab  group | Chemotherap**y** group |
| PFS |  |  |  |  |
| Weibull | 1055.68 | 992.70 | 1062.50 | 999.47 |
| Log-logistic | 1027.27 | 990.30 | 1034.08 | 997.08 |
| Log-normal | 1041.69 | 1031.02 | 1048.50 | 1037.79 |
| Gompertz | 1082.23 | 1036.66 | 1089.04 | 1043.43 |
| Exponential | 1086.61 | 1117.69 | 1090.02 | 1121.08 |
| Gamma | 1045.27 | 992.69 | 1052.09 | 999.47 |
| OS |  |  |  |  |
| Weibull | 1328.37 | 1354.37 | 1335.18 | 1361.15 |
| Log-logistic | 1309.85 | 1338.27 | 1316.67 | 1345.05 |
| Log-normal | 1324.70 | 1347.44 | 1331.51 | 1354.21 |
| Gompertz | 1353.75 | 1382.66 | 1360.57 | 1389.44 |
| Exponential | 1368.24 | 1409.52 | 1371.65 | 1412.91 |
| Gamma | 1320.95 | 1345.76 | 1327.77 | 1352.54 |

AIC: Akaike information criterion; BIC: Bayesian Information Criterion; OS: Overall survival; PFS: Progression-free survival;

**
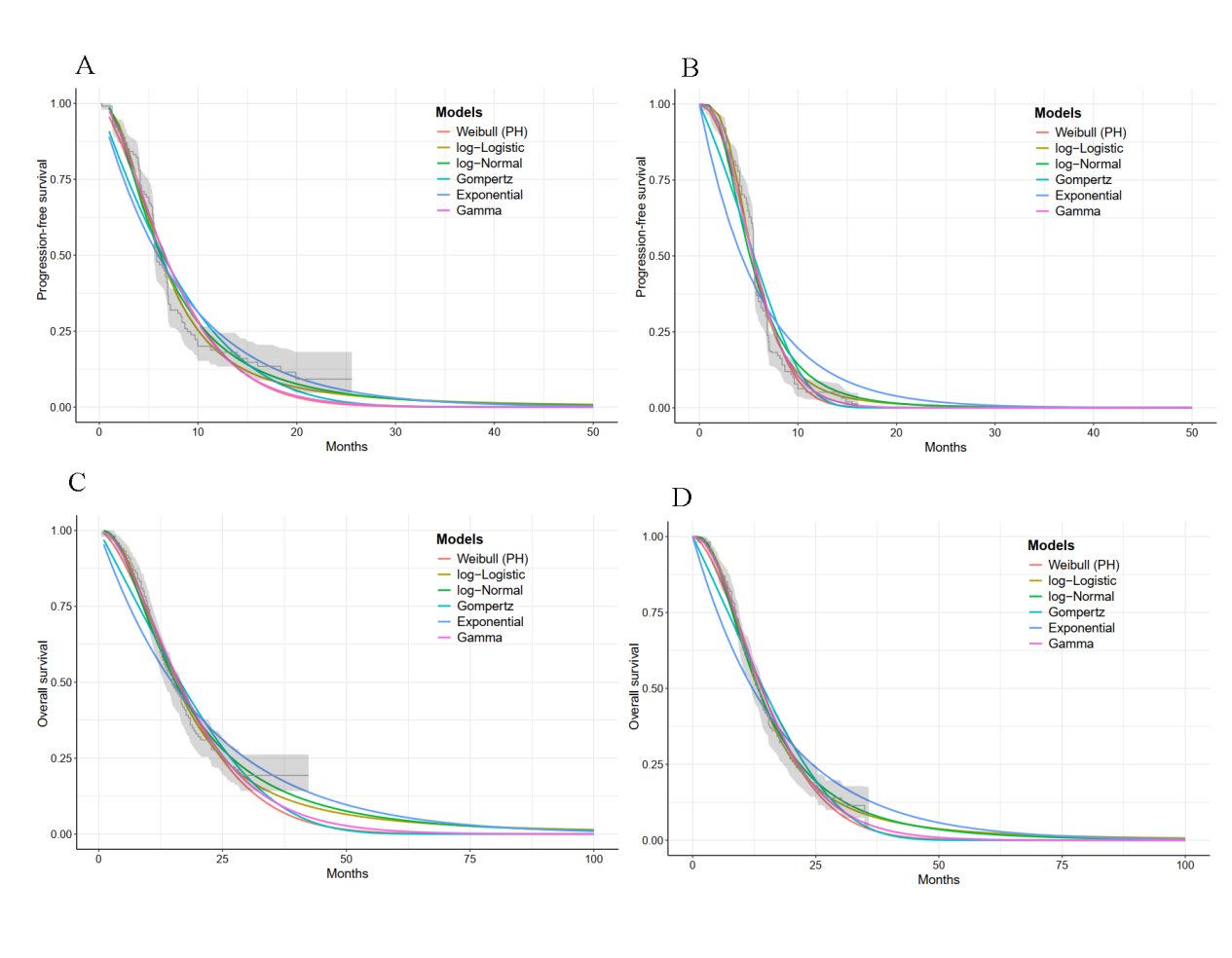
Supplementary Figure 1.** A:Modes simulation visual progression-free survival curve of toripalimab group;B:Modes simulation visual progression-free survival curve of chemotherapy group;C:Modes simulation visual overall survival curve of toripalimab group;D: Modes simulation visual overall survival curve of chemotherapy group
